# Receipt of evidence-based brief cessation interventions by health professionals and use of cessation assisted treatments among current adult cigarette-only smokers: National Adult Tobacco Survey, 2009–2010

**DOI:** 10.1186/s12889-016-2798-2

**Published:** 2016-02-11

**Authors:** Judy Kruger, Alissa O’Halloran, Abby C. Rosenthal, Stephen D. Babb, Michael C. Fiore

**Affiliations:** Office on Smoking and Health, Centers for Disease Control and Prevention, National Center for Chronic Disease Prevention and Health Promotion, Atlanta, GA 30341 USA; Contractor (NGIS) for Office on Smoking and Health, National Center for Chronic Disease Prevention, Atlanta, GA 30341 USA; Health Systems Consulting, Atlanta, GA 30341 USA; Center for Tobacco Research and Intervention, University of Wisconsin School of Medicine and Public Health, Madison, WI 53711 USA; Office on Smoking and Health, Centers for Disease Control and Prevention, 4770 Buford Highway, N.E., F-79, Atlanta, GA 30341-3724 USA

**Keywords:** Smoking cessation, USPHS clinical guideline, Tobacco, Health professionals, Clinicians

## Abstract

**Background:**

Helping tobacco smokers to quit during a medical visit is a clinical and public health priority. Research suggests that most health professionals engage their patients in at least some of the ‘5 A’s’ of the brief cessation intervention recommended in the U.S. Public Health Service Clinical Practice Guideline, but information on the extent to which patients act on this intervention is uncertain. We assessed current cigarette-only smokers’ self-reported receipt of the 5 A’s to determine the odds of using optimal cessation assisted treatments (a combination of counseling and medication).

**Methods:**

Data came from the 2009**–**2010 National Adult Tobacco Survey (NATS), a nationally representative landline and mobile phone survey of adults aged ≥18 years. Among current cigarette-only smokers who visited a health professional in the past 12 months, we assessed patients’ self-reported receipt of the 5 A’s, use of the combination of counseling and medication for smoking cessation, and use of other cessation treatments. We used logistic regression to examine whether receipt of the 5 A’s during a recent clinic visit was associated with use of cessation treatments (counseling, medication, or a combination of counseling and medication) among current cigarette-only smokers.

**Results:**

In this large sample (*N* = 10,801) of current cigarette-only smokers who visited a health professional in the past 12 months, 6.3 % reported use of both counseling and medication for smoking cessation within the past year. Other assisted cessation treatments used to quit were: medication (19.6 %); class or program (3.8 %); one-on-one counseling (3.7 %); and telephone quitline (2.6 %). Current cigarette-only smokers who reported receiving all 5 A’s during a recent clinic visit were more likely to use counseling (odds ratio [OR]: 11.2, 95 % confidence interval [CI]: 7.1–17.5), medication (OR: 6.2, 95 % CI: 4.3–9.0), or a combination of counseling and medication (OR: 14.6, 95 % CI: 9.3–23.0), compared to smokers who received one or none of the 5 A’s components.

**Conclusions:**

Receipt of the ‘5 A’s’ intervention was associated with a significant increase in patients’ use of recommended counseling and medication for cessation. It is important for health professionals to deliver all 5 A’s when conducting brief cessation interventions with patients who smoke.

## Background

In the United States, approximately 480,000 people die from a smoking-related illness each year [1]. Smoking cessation can significantly reduce the risk of developing smoking-related diseases and increase life expectancy [1, 2]. A United States Public Health Service (USPHS) Clinical Practice Guideline emphasizes the importance of health professionals providing tobacco dependence treatment to their patients [3]. The Guideline recommends that health professionals follow a brief, evidence-based cessation intervention known as the ‘5 A’s’: Ask about tobacco use, Advise tobacco users to quit, Assess willingness to make a quit attempt, Assist tobacco users in making a quit attempt, and Arrange for follow-up. The Guideline also notes that tobacco dependence treatments are highly cost-effective [3–5]. Although effective clinical interventions exist, patient-reported data suggest that health professionals do not consistently deliver evidence-based cessation treatments to patients who smoke [3, 6].

Quitting smoking is difficult and often requires multiple quit attempts, so it is important for health professionals to repeatedly address cessation with their patients who smoke [7]. As part of the 5 A’s, the USPHS Clinical Practice Guideline recommends that health professionals routinely provide brief counseling and recommend medications (unless contraindicated) for tobacco cessation. Although the combination of counseling and medication is more effective for smoking cessation than either counseling or medication alone, smokers’ use of this combined approach is limited [8, 9]. Collectively, these data underscore the importance of health professionals and the health systems in which they work, to deliver all five of the 5 A’s at every clinic visit [3].

Health professionals have frequent contact with their patients, have high credibility, and play an important role in educating their patients about the consequences of smoking [3, 7]. However, no recent studies have examined the extent to which patients actually use cessation treatments recommended during the medical encounter. While reports suggest delivery of the 5 A’s intervention yields greater patient use of cessation services, many health professionals do not routinely provide all of these components [7]. Thus, more in-depth information on patient-reported receipt of the 5 A’s and how this affects patients’ use of cessation assisted treatments may help guide efforts to increase health professionals’ delivery of all components of this evidence-based intervention. To address this gap in the literature, this study assessed the association between smokers’ self-reported receipt of the 5 A’s and use of cessation assisted treatments, including the optimal recommended combination of counseling and medication.

## Methods

### Data source

The 2009–2010 National Adult Tobacco Survey (NATS) was a stratified, national landline and mobile phone survey of noninstitutionalized civilian adults aged 18 years or older residing in the 50 U.S. states and the District of Columbia. The study design has been described in detail elsewhere [10] and the data used for the study is openly available. Respondent selection varied by phone type. For landline numbers, one adult was randomly selected from each eligible household. In contrast, adults who only used mobile phones were selected through screening of a sample of mobile phone numbers. In total, 118,581 NATS interviews were completed (landline *n* = 110,634, mobile phone *n* = 7,947) in both English and Spanish from October 20, 2009 to February 28, 2010. The national Council of American Survey and Research Organizations response rate, or the number of completed interviews divided by the number of eligible respondents in the sample, was 37.6 % (landline = 40.4 %, mobile phone = 24.9 %) [11]. The national cooperation rate, or the number of completed interviews divided by the number of eligible respondents who were successfully reached by an interviewer, was 62.3 % (landline = 61.9 %, mobile phone = 68.7 %).

**Fig. 1:**
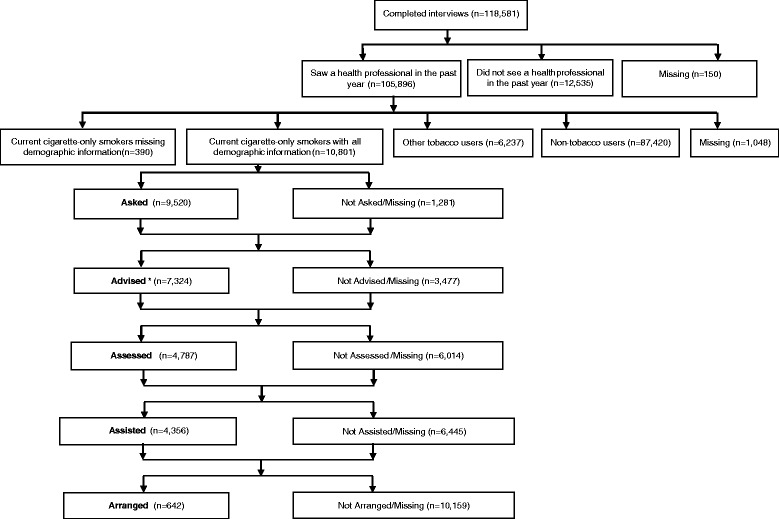
Schematic of participant inclusion and exclusion—National Adult Tobacco Survey, 2009–2010

Of the 118,581 respondents who completed interviews, a total of 105,896 respondents reported seeing a health care provider in the past year (Fig. 1). Of these, 10,801 were current cigarette-only smokers who had seen a health professional in the past 12 months and who provided complete demographic information (sex, age, race/ethnicity, and education). These 10,801 current cigarette smokers served as our study sample and were asked questions related to provider delivery of the 5 A’s: Ask, Advise, Assess, Assist, and Arrange (Appendix) and are presented in the standard 5 A’s order.

### Measures

#### Smoking status

Cigarette smoking status was assessed using two standard questions. The first question was, “Have you smoked at least 100 cigarettes in your entire life?” Those who responded yes were then asked, “Do you now smoke cigarettes every day, some days, or not at all?” Respondents who stated that they currently smoked every day or some days were considered to be a current cigarette smoker. The study assessed current smokers who did not use any other tobacco products (cigarette-only smokers).

#### Receipt of brief cessation interventions

To determine whether respondents visited a health professional, adults who smoked were asked, “In the past 12 months, have you seen a doctor, dentist, nurse, or other health professional?” Cigarette-only smokers who had visited a health professional in the past 12 months were asked about receipt of all of the 5 A’s from their health professional. To determine if a health professional ‘Asked’ about tobacco use, respondents were asked, “In the past 12 months, did any doctor, dentist, nurse, or other health professional ask if you smoke cigarettes or use any other tobacco products?” To determine if a health professional ‘Advised’ them to quit, respondents were asked, “In the past 12 months, did any doctor, dentist, nurse, or other health professional advise you to quit smoking cigarettes or using any other tobacco products?” To determine if a health professional ‘Assessed’ willingness to make a quit attempt, respondents were asked, “The last time a health professional advised you to quit using tobacco, did they also ask if you wanted to try to quit?” To determine if a health professional ‘Assisted’ respondents in quitting, they were asked, “The last time a health professional advised you to quit using tobacco, did they also offer any assistance, information, or additional advice to help you quit?” To determine if a health professional ‘Arranged’ or scheduled a follow-up contact, they were asked, “(Did they) schedule any follow-up contacts, either in person or by phone, or arrange for someone else to call you to see how your quit attempt was going?” Response options for each question were yes, no, don’t know/not sure, or refused.

Based on the skip pattern used in the NATS, not all smokers were asked about receipt of all of the 5 A’s. The NATS questionnaire first queried smokers regarding whether they were ‘Advised’ to quit. A total of 7,324 smokers reported that they received such advice (*n* = 3,477 were not advised or missing). The NATS survey followed with a question on ‘Asked’ (asking about tobacco use) which was queried of the 3,477 who responded no to the ‘Advised’ question. Of these, 2, 196 reported that they were ‘Asked’ and 1,281 were not asked or were missing. Because we assumed that only smokers were ‘Advised’ to quit, those who reported yes to the Advised question were recoded as responding yes to the ‘Asked’ question. This resulted in an estimated 9,520 (7,324 + 2,196) respondents reporting that they were ‘Asked’ about their tobacco use. Among the 7,324 who were ‘Advised’ to quit, a total of 4,787 reported yes to the ‘Assessed’ question (asking about willingness to quit). Those who were ‘Advised’ were also asked the ‘Assist’ question (asked if received assistance with quitting); 4,356 respondents reported that they were ‘Assisted’ with quitting. Those who were ‘Assisted’ were then asked the ‘Arrange’ question (asked if follow-up was arranged); 642 responded yes to the ‘Arrange’ question. On the NATS, the actual word order for the 5 A’s were: Advised, Asked, Assessed, Assisted, and Arranged; however, we present findings in the standard 5 A’s order (Fig. 1). To assess self-reported delivery of the 5 A’s, we limited the scope to cigarette-only smokers who saw a health professional in the past 12 months to examine patient-reported receipt of the 5 A intervention among those eligible. We used the same denominator (*n* = 10,801) for each of the 5 A items.

#### Receipt of specific “Assisted” interventions

Respondents who said they had been offered assistance (‘Assist’) were also asked about the provision of specific forms of assistance: “The last time a health professional advised you to quit using tobacco, did they provide you with booklets, videos, website addresses, or other information to help you quit?”; “Did they put you in contact with, or tell you how to contact, a telephone quitline, a class or program, or one-on-one counseling?”; “(Did they) recommend or prescribe a nicotine patch, nicotine gum, lozenges, nasal spray, an inhaler, or pills such as Wellbutrin, Zyban, Bupropion, Chantix, or Varenicline?”; and “Did they help you set a specific date to quit using tobacco products?”

#### Cessation treatment use

Respondents were asked four questions about their use of cessation resources to help them quit: “Did you call a telephone quitline?”; “Did you use a class or program to help you quit?”; “Did you use one-on-one counseling from a health professional to help you quit?”; and “Did you use any of the following medications: a nicotine patch, nicotine gum, nicotine lozenges, nicotine nasal spray, a nicotine inhaler, or pills such as Wellbutrin, Zyban, Bupropion, Chantix, or Varenicline to help you quit?” Those who answered yes to one of these questions were identified as having used that cessation treatment. Respondents who reported calling a telephone quitline, using a class or a program, or using one-on-one counseling were aggregated into a single ‘counseling’ variable. Respondents who reported using any of the medications were classified as using medication. Respondents who reported using ‘counseling’ and any cessation medication were classified as using the combination of counseling and medication.

#### Respondent characteristics

The following respondent characteristics were included in the analysis: sex, age (18–24, 25–34, 35–54, or ≥55 years), race/ethnicity (non-Hispanic white, non-Hispanic black, Hispanic, or non-Hispanic other), and education (less than high school, high school diploma, some college, college educated). The race/ethnicity ‘non-Hispanic other’ category included respondents who were American Indian or Alaska Native, Native Hawaiian or Pacific Islander, Asian, multiracial, or some other race.

### Statistical analysis

Descriptive analyses were conducted overall and among current cigarette-only smokers by sex, age, race/ethnicity, and educational attainment. We estimated the prevalence of using counseling, medication, or a combination of counseling and medication by receipt of the 5 A’s and demographic characteristics. We conducted multivariate logistic regression analyses to estimate the association between receipt of the 5 A’s and use of cessation services (counseling, medication, or combination of counseling and medication) while adjusting for sex, age, race/ethnicity, and educational attainment. Three separate logistic regression models were constructed with use of each of the cessation services as the dependent variable, and receipt of 5 A’s and demographic variables as the independent variables. Odds ratios (ORs) with 95 % confidence intervals (CIs) were calculated. SAS-callable SUDAAN (version 9.2; Research Triangle Institute, Research Triangle Park, NC) was used to accommodate the complex sampling design. Landline data were weighted according to the selection probability of the telephone number, the number of adults in the household, and the number of landline telephone numbers in the household. Mobile phone data were weighted only according to the selection probability of the mobile phone number because a mobile phone was assumed to be used only by the person who answered [12].

## Results

### Sample characteristics

Table 1 presents weighted data on demographic characteristics of the overall sample of current cigarette-only smokers (*n* = 10,801). Among adult current cigarette-only smokers, most were between 35 and 54 years of age (44.8 %), non-Hispanic white (74.0 %), and had a high school diploma (34.8 %).

**Table 1:** Characteristics of current cigarette-only smokers who visited a health professional within the past 12 months

	Current cigarette-only smokers^a^		
	(*N* = 10,801)		
Characteristic	n	%^b^	95 % CI
Sex			
Men	3935	45.3	43.4–47.3
Women	6866	54.7	52.7–56.6
Age (years)			
18–24	456	9.9	8.6–11.2
25–34	1432	21.7	19.9–23.4
35–54	4580	44.8	43.0–46.7
55+	4333	23.6	22.2–25.0
Race/ethnicity			
White, non-Hispanic	8783	74.0	72.1–76.0
Black, non-Hispanic	915	10.8	9.6–12.1
Hispanic	366	9.5	7.8–11.1
Other, non-Hispanic	737	5.6	4.8–6.4
Education			
< High school	1191	20.3	18.4–22.2
High school diploma	3182	34.8	33.1–36.6
Some college	4007	32.8	31.2–34.5
≥ College	2421	12.1	11.2–12.9

^a^Current cigarette-only smokers who were surveyed about whether a health care professional had provided any of the ‘5 A’s’ brief counseling intervention

^b^Percentages are weighted for age, sex race/ethnicity, marital status, and education

### Receipt of brief cessation interventions

Among current cigarette-only smokers who had seen a health professional in the last 12 months, 88.3 % were ‘Asked’ about current tobacco use, 66.4 % were ‘Advised’ to quit, 43.4 % were ‘Assessed’ for their willingness to quit, 38.6 % were ‘Assisted’, and 6.3 % reported that their health professional ‘Arranged’ a follow-up (Table 2). Rates of receipt of ‘Assisted’ interventions from a health professional were: cessation medication (24.9 %); cessation materials (booklets, videos, or website addresses) (24.4 %); referral to counseling (a telephone quitline, a class or program, or one-on-one counseling) (17.8 %); referral to counseling and prescribing medication (11.2 %); and helping patients set a date to quit (5.6 %).

**Table 2:** Receipt of brief cessation intervention by current cigarette-only smokers — National Adult Tobacco Survey, 2009–2010

Brief cessation intervention^a^	Number	Percent^b^
Did the health care professional …		
ASK about current tobacco use		
Yes	9520	88.3
No^c^	1281	11.7
ADVISE to quit		
Yes	7324	66.4
No^c^	3477	33.6
ASSESS willingness to quit now		
Yes	4787	43.4
No^c^	6014	56.6
ASSIST by providing appropriate tobacco dependence treatment and intervene to increase motivation to quit^d^		
Yes	4356	38.6
No^c^	6445	61.4
Provide specific ‘Assist’ options		
Cessation medications^e^	2835	24.9 %
Cessation materials^f^	2645	24.4 %
Counseling^g^	1934	17.8 %
Counseling and medication^h^	1269	11.2 %
Set a quit date^i^	586	5.6 %
ARRANGE follow-up		
Yes	642	6.3
No^c^	10159	93.7

Note: Among all current cigarette-only smokers who reported seeing a doctor, dentist, nurse or other health professional in the past 12 months

^a^Data are presented in the standard 5A order

^b^Percentage is weighted for age, sex race/ethnicity, and education. Weighted percentage refers to respondents who answered yes to using the selected cessation treatments

^c^Refers to respondents who answered no, don’t know/not sure, refused, or didn’t respond to the question

^d^Numbers for specific ‘Assisted’ interventions listed may not add to 38.6 % because multiple options could be selected

^e^Cessation medications refers to a nicotine patch, nicotine gum, lozenges, nasal spray, an inhaler, Wellbutrin, Zyban, Bupropion, Chantix, or Varenicline

^f‘^Cessation materials refers to booklets, videos, or Web site addresses

^g^Counseling refers to referral to a telephone quitline, a class or program, or one-on-one counseling

^h^Refers to the mutually exclusive combination of counseling and cessation medication

^i^Set a quit date refers to a health professional helping to set a date to quit

### Cessation treatment use

**Fig. 2:**
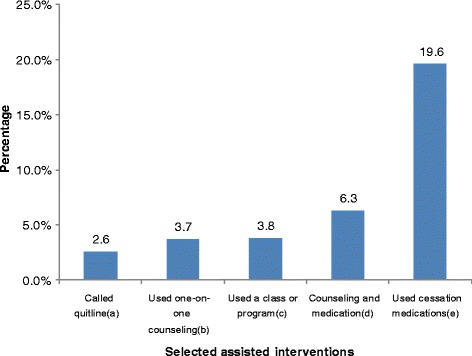
Use of tobacco cessation treatments by current cigarette-only smokers—NATS, 2009–2010

Among current cigarette-only smokers, 19.6 % reported using medication, 6.3 % reported use of a combination of counseling and medication, 3.8 % reported using a class or program, 3.7 % reported using one-on-one counseling, and 2.6 % reported calling a telephone quitline (Fig. 2).

### Receipt of the 5 A’s intervention and use of cessation assisted treatment

Among respondents who received all 5 A’s, 31.7 % reported using counseling compared with only 3.8 % who received one or none of the 5 A’s (Table 3). Logistic regression analyses indicated that respondents who received all 5 A’s had higher odds of using counseling services (OR: 11.2, 95 % CI: 7.1–17.5) compared to those who received one or none of the 5 A’s. Respondents who received any four (OR: 2.4, 95 % CI: 1.6–3.5) or any three (OR: 1.8, 95 % CI: 1.2–2.9) of the 5 A’s had higher odds of using counseling services compared to those who received one or none of the 5 A’s. Among current cigarette-only smokers, odds of using counseling services were higher among respondents aged 35–54 years (OR: 2.9, 95 % CI: 1.6–5.3) and those aged ≥55 years (OR: 2.6, 95 % CI: 1.4–4.8) than those aged 18–24 years.

**Table 3:** Adjusted odds of using cessation assisted treatment among U.S. adults^a^ who received 5A’s interventions—National Adult Tobacco Survey, 2009–2010

	Counseling^b^		Medication^c^		Combination of counseling and medication^d^	
	% (95 % CI)	OR^e^ (95 % CI)	% (95 % CI)	OR^e^ (95 % CI)	% (95 % CI)	OR^e^ (95 % CI)
5A’s						
Received all 5	31.7 (24.7–39.5)^f^	11.2 (7.1–17.5)^f^	46.8 (38.3–55.4)^f^	6.2 (4.3–9.0)^f^	29.0 (22.3–36.8)^f^	14.6 (9.3–23.0)^f^
Received any 4	9.7 (7.9–11.7)^f^	2.4 (1.6–3.5)^f^	25.3 (22.6–28.3)^f^	2.2 (1.7–2.8)^f^	8.2 (6.6–10.1)^f^	2.9 (2.0–4.4)^f^
Received any 3	7.2 (5.2–9.9)^f^	1.8 (1.2–2.9)^f^	20.8 (16.8–25.5)^f^	1.8 (1.3–2.5)^f^	5.5 (3.8–7.9)^f^	2.0 (1.3–3.3)^f^
Received any 2	4.6 (3.2–6.6)	1.2 (0.7–1.9)	14.3 (11.7–17.4)	1.1 (0.8–1.5)	3.1 (2.0–4.8)	1.2 (0.7–2.0)
Received any 1 or 0	3.8 (2.8–5.1)	Ref	12.4 (10.5–14.6)	Ref	2.6 (1.9–3.5)	Ref
Sex						
Men	6.8 (5.6–8.4)	Ref	18.2 (16.0–20.7)	Ref	5.3 (4.2–6.6)	Ref
Women	8.5 (7.4–9.8)	1.2 (0.9–1.6)	20.8 (19.1–22.7)	1.1 (0.9–1.4)	7.1 (6.0–8.3)^f^	1.3 (1.0–1.8)
Age (years)						
18–24	2.9 (1.7–5.0)	Ref	10.4 (7.2–14.7)	Ref	^g^	Ref
25–34	5.4 (3.8–7.5)^f^	1.7 (0.8–3.5)	17.2 (14.3–20.7)^f^	1.5 (1.0–2.5)	3.7 (2.6–5.2)	1.9 (0.8–4.3)
35–54	9.5 (8.0–11.1)^f^	2.9 (1.6–5.3)^f^	23.2 (20.9–25.6)^f^	2.1 (1.4–3.3)^f^	7.9 (6.6–9.5)	4.0 (1.9–8.8)^f^
55+	8.8 (7.2–10.6)^f^	2.6 (1.4–4.8)^f^	19.0 (16.9–21.4)^f^	1.6 (1.0–2.5)^f^	7.4 (5.9–9.2)	3.5 (1.7–7.6)^f^
Race/ethnicity						
White, non-Hispanic	7.8 (6.9–8.9)	Ref	20.7 (19.2–22.4)	Ref	6.4 (5.6–7.4)	Ref
Black, non-Hispanic	8.1 (5.4–12.1)	1.0 (0.6–1.7)	16.7 (12.8–21.5)	0.7 (0.5–1.0)	5.6 (3.6–8.4)	0.8 (0.5–1.3)
Hispanic	^g^	0.6 (0.3–1.2)	13.2 (8.0–20.9)^f^	0.6 (0.4–1.1)	^g^	0.7 (0.3–1.5)
Other, non-Hispanic	11.9 (8.0–17.3)	1.5 (1.0–2.4)	21.6 (16.2–28.1)	0.9 (0.7–1.4)	9.2 (5.8–14.3)	1.4 (0.8–2.4)
Education						
< High school	7.3 (5.3–9.9)	Ref	16.4 (12.8–20.7)	Ref	6.1 (4.3–8.6)	Ref
High school diploma	7.4 (5.8–9.3)	1.1 (0.7–1.7)	17.7 (15.6–20.1)	1.1 (0.8–1.6)	5.8 (4.5–7.4)	1.0 (0.6–1.7)
Some college	8.4 (7.0–9.9)	1.1 (0.8–1.7)	23.6 (21.3–26.2)^f^	1.6 (1.1–2.2)^f^	6.8 (5.5–8.3)	1.1 (0.7–1.8)
≥ College	8.2 (6.5–10.2)	1.21 (0.8–1.9)	19.8 (16.9–22.9)	1.3 (0.9–1.8)	6.6 (5.1–8.6)	1.2 (0.7–1.9)

Note: Among current cigarette-only smokers who received each sub-set of the 5A’s, the overall percentage for those who received any one or none of the 5A’s was 33.6 % (31.8–35.5); any two was 18.3 % (16.9–19.7); any three was 13.9 % (12.6–15.3); any four was 28.1 % (26.5–29.7); and all five was 6.1 % (5.0–7.1)

^a^Current cigarette-only smokers who had seen a health professional in the past 12 months

^b^Counseling refers to individual, group, or telephone quitline counseling

^c^Medication refers to nicotine patch, nicotine gum, nicotine lozenges, nicotine nasal spray, a nicotine inhaler, or pills

^d^Prevalence of use of counseling and medication

^e^Adjusted odds ratios. Logistic regression compared those who reported receiving all 5 A’s, any 4 A’s, any 3 A’s, any 2 A’s with those who reported receiving only 1 or 0 A’s

^f^Items are statistical significance (*p* < 0.05)

^g^Estimate may not be reliable due to relative standard error >30 %

Among respondents who received all 5 A’s, 46.8 % reported using cessation medication, compared with only 12.4 % who received one or none of the 5 A’s. Logistic regression analyses indicated that respondents who received all 5 A’s had higher odds of using cessation medication (OR: 6.2, 95 % CI: 4.3–9.0) compared to those who received one or none of the 5 A’s. Respondents who received any four (OR: 2.2, 95 % CI: 1.7–2.8) or any three (OR: 1.8, 95 % CI: 1.3–2.5) of the 5 A’s had an increased odds of using cessation medications compared to those who received one or none of the 5 A’s. Odds of using medication to try to quit were higher among respondents aged 35–54 years (OR: 2.1, 95 % CI: 1.4–3.3) and those aged ≥55 years (OR: 1.6, 95 % CI: 1.0–2.5) than those aged 18–24 years. Odds of using medication were higher among respondents with some college education (OR: 1.6, 95 % CI: 1.1–2.2) than those without a high school degree.

Among respondents who received all 5 A’s, 29.0 % reported using a combination of counseling and medication compared with only 2.6 % who received one or none of the 5 A’s. Logistic regression analyses indicated that respondents who received all 5 A’s had higher odds of using a combination of counseling and medication (OR: 14.6, 95 % CI: 9.3–23.0) compared to those who received one or none of the 5 A’s. Respondents who received any four (OR: 2.9, 95 % CI: 2.0–4.4) or any three (OR: 2.0, 95 % CI: 1.3–3.3) of the 5 A’s had higher odds of using a combination of counseling and medication compared to those who received one or none of the 5 A’s. Odds of using a combination of counseling and medication to try to quit were higher among respondents aged 35–54 years (OR: 4.0, 95 % CI: 1.9–8.4) and those aged ≥55 years (OR: 3.5, 95 % CI: 1.7–7.6), than those 18–24 years of age.

Among respondents who received each sub-set of the 5A’s, the proportion who received any one or none of the 5A’s was 33.6 %, any two was 18.3 %, any three was 13.9 %, any four was 28.1 %, or all five was 6.1 % (data not shown).

## Discussion

Current cigarette-only smokers who reported receiving the full 5 A’s intervention (Ask, Advise, Assess, Assist, Arrange) were approximately 15 times as likely to report using the combination of counseling and medication (optimal treatment recommended by the USPHS Clinical Practice Guideline) as those who received one or none of the 5 A’s. Additionally, we found that smokers who received any three or four components of the 5 A’s were more likely to use cessation treatment compared to smokers who received one or none of the 5 A’s. Among current cigarette-only smokers who received all 5 A’s, 29.0 % reported using a combination of counseling and medication compared with 8.2 % who received four of the 5 A’s. These findings suggest that delivery of all five elements of the 5 A’s intervention is associated with greater use of cessation treatments. Moreover, findings suggest that any four or three of 5A’s could also significantly promote cessation treatments compared to one or none of 5A’s. The ABC pathway (Ask, Brief advice, Cessation) from New Zealand incorporates the 5 A’s into three steps and emphasizes the important role health professionals play in offering tobacco users guidance to access cessation support [13]. However the importance of delivering all 5 A’s is relevant, given data suggesting that a large proportion of health professionals now deliver the first 2 A’s (Ask, Advise) but that a much lower percentage deliver the final two A’s (Assist, Arrange) [6, 14–17]. This may be because ‘Assisting’ with cessation and ‘Arranging’ follow-up are often time consuming, may not be reimbursed, and require effective communication skills to tailor the intervention to patients’ needs [18–20]. Our findings highlight the potential benefits to smokers when health professionals adopt the full complement of recommended evidence-based cessation treatments [3].

In this large sample of current cigarette-only smokers who reported seeing a health professional within the last year, a marginal number reported being 'Assessed' for their willingness to quit or 'Assisted' by offer of cessation treatments, such as a telephone quitline. However, the finding that a minority of current smokers recalled having received the 'Assist' and 'Arrange' component of the 5 A’s is consistent with previous studies on patient use of tobacco dependence treatments [15, 16, 21], and suggests the need for continued efforts to expand health professionals’ implementation of brief advice and cessation support. The 'Assess' step is key to determining the best approach to take for 'Assist' and what should be done about 'Arrange'. To enhance cessation, at each office visit after advising the smoker to quit, health professionals should 'Assess' each smoker’s willingness to make a quit attempt and tailor the 'Assist' and 'Arrange' components to address the smoker’s readiness to quit [3]. Given that providers who are proactive in ‘Assisting’ patients to use evidence-based tobacco cessation treatments have a significant impact on long-term quit rates [22], health professionals may be most effective in helping their patients quit smoking by providing assistance and arranging follow-up for smokers to maximize quit attempts, treatment use, and quit rates [23, 24]. This study also noted that patient participation in any of the ‘Assist’ strategies (counseling, medication, combination of counseling and medication) was higher in those aged 35 or older than those aged 18–24. The finding that older patients are more likely to use cessation assisted treatment could be related to health professionals spending more time with this population in efforts to improve patient adherence to treatment; but may also be due to the fact that older patients often have a variety of chronic health conditions and demonstrate a greater interest in quitting [19]. Further delivery of tobacco education including media campaigns such as the CDC Tips from Former Smokers campaign can motivate youth tobacco users to try to quit and to seek information on quitting [25]. Population-based strategies including providing telephone cessation counseling [25] can motivate tobacco users to quit while simultaneously making evidence-based cessation treatments readily available particularly to those who are vulnerable to social and environmental influences of cigarette use [26]. Since most cigarette smokers are receptive to their physicians’ advice and willing to discuss quitting smoking [3], our findings suggest that opportunities exist for health professionals to improve delivery of tobacco cessation assistance, including counseling and provision of medications, to increase patient use of these strategies.

The Institute of Medicine found that helping tobacco users to quit is essential to reduce tobacco use [27]. Our finding that 38.6 % of NATS current cigarette smokers reported receiving ‘Assistance’ to quit highlights the opportunity for health professionals to expand delivery of the ‘Assist’ brief intervention step [17]. We also found that less than 25 % of respondents reported receipt of any specific ‘Assisted’ strategy (cessation medication, cessation materials, counseling, counseling and medication, set a quit date) from a health professional. A number of factors are responsible for the lack of consistent delivery of brief cessation interventions, including time constraints, lack of expertise, lack of financial incentives, fear of alienating patients, and skepticism about smokers being able to quit [28]. The 2008 USPHS Guidelines cite a number of health system barriers that may impede clinicians’ assessment and treatment of smokers, including inadequate institutional support for routine assessment and treatment of tobacco use, a lack of insurance coverage for tobacco use treatment, or inadequate payment for treatment [3]. Health system changes that integrate cessation interventions into routine clinical care have been found to increase the likelihood that health professionals consistently screen patients for tobacco use and intervene with patients who use tobacco, thereby making evidence-based tobacco dependence treatment the standard of care and increasing cessation [3, 5, 29].

We found that use of the both counseling and medication treatment components, which is the optimal approach recommended by the USPHS Clinical Practice Guideline [3] to help smokers quit, was considerably higher among current smokers who recalled having received such counseling and medication during a medical encounter. Because tobacco dependence is a chronic condition that often requires multiple cessation attempts, primary care providers must repeatedly address cessation with their patients who use tobacco. The U.S. Preventive Services Task Force (USPSTF) is an independent, volunteer panel of national experts on prevention and evidence-based medicine. Their role is to make recommendations based on the body of peer-reviewed evidence about clinical preventive services and indicate the quality of the evidence using one of five letter grades (A, B, C, D, or I) [4]. The USPSTF assigns a letter grade A or B to recommend a preventive service where there is high certainty that the net benefit is moderate to substantial. Medicare, and a number of state Medicaid programs have recently expanded coverage of cessation treatments [30, 31]. In addition, several provisions in the 2010 Affordable Care Act expanded private and Medicaid cessation coverage. More specifically, the legislation requires all non-grandfathered private plans to cover with no cost sharing preventive services that receive an A or B rating from the USPSTF, which includes tobacco cessation interventions [32]. In May 2014, the United States Departments of Labor, Treasury, and Health and Human Services released a guidance document that provided specificity regarding the nature of Affordable Care Act tobacco cessation coverage – that it must include at least two quit attempts per year with coverage for both cessation counseling and medication [33]. Moreover, as of October 2010, the Affordable Care Act requires state traditional Medicaid programs to cover a comprehensive cessation benefit for pregnant women [32]. Effective January 2014, the legislation also bars states from excluding FDA-approved cessation medications from their coverage for all traditional Medicaid enrollees [32]. In sum, recent insurance policy changes may increase the capacity of clinicians to help their patients quit [32].

Systems-level interventions can facilitate the delivery of all of the 5 A’s and increase cessation [5, 29]. Such interventions include the use of provider reminder systems, which prompt health professionals to assess smoking status during each medical visit and to intervene with patients who smoke [3, 26, 34]. Another example of a systems intervention is electronic health records, which can help health professionals monitor patients’ smoking status and support delivery of evidence-based cessation interventions and referrals [35]. Strategic efforts to reconfigure policies and systems to increase delivery of cessation services in the health care setting may include the use non-physician staff to administer some of the 5 A’s (e.g., Asking, Assisting, or Arranging) [5, 23]. Reimbursement of clinicians for making counseling and other cessation treatment a routine part of care may also support delivery of evidence-based cessation interventions [3, 24].

This study has several limitations. First, although the dataset was large and representative of the U.S. population, these data reflect patients’ self-reported receipt and use of cessation interventions from a health professional, which may be subject to recall bias. Additional research that examines provider behavior through a monitoring system, electronic medical record, or direct observation of the medical encounter may be beneficial. Second, these data are cross-sectional, and thus, it was not possible to assess causal relationships between provider delivery of the 5 A’s and patient use of cessation treatments. Third, questionnaire design did not allow estimates to be categorized by health professional type; therefore, we could not examine provision of tobacco cessation interventions by different health professions (i.e., doctor, dentist, nurse, or other health professional). Fourth, the study questions asked whether the 5 A behaviors had occurred in any visit in the past year, and it is not possible to determine the timeframe for cessation assisted treatment use, or how frequently multiple behaviors occurred in any given encounter. Fifth, the question order of the 5 A’s was not asked in the typical traditional format and do not fully align with measures used elsewhere in the literature. While the NATS survey included all of the 5 A’s, because of the skip pattern, it was not possible to determine if the question order of all of the 5A’s impacted cessation treatment use. Moreover, misclassification bias could have occurred because those who were never asked an item due to the skip pattern or answered ‘don’t know’ or refused were included in the denominator. Sixth, analyses do not include ever smokers who may have visited a health professional in the past 12 months but who quit more than 30 days before the survey was administered. Consequently, recall bias may exist as uptake of the 5 A’s (receipt of cessation interventions among smokers interested in quitting) among then smokers with an ongoing quit attempt lasting 30 days or more was not measured.

## Conclusions

We demonstrate for the first time that cigarette-only smokers who receive all of the 5 A’s during a medical encounter are more likely to use counseling and medication to quit, compared to smokers who receive one or none of the 5 A’s. Among smokers who reported receiving any cessation assistance during a recent clinic visit, the most common treatment used was cessation medication, followed by the combination of counseling and medication, a class or program, one-on-one counseling, and calling a telephone quitline. Given that patients who receive all five of the 5 A’s are 15 times more likely to report using the most effective cessation treatment (medication and counseling), whenever feasible health professionals should be encouraged to deliver all 5 A’s with patients who smoke. Such efforts may help maximize treatment use.

## Appendix

Very brief U.S. Public Health Service Clinical Practice Guideline ‘5 A’s’ intervention and related National Adult Tobacco Survey questions.

1: Ask: Identify all tobacco users.

Question: In the past 12 months, did any doctor, dentist, nurse, or other health professional ask if you smoke cigarettes or use any other tobacco products?

2: Advise: Urge or advise tobacco users to quit.

Question: In the past 12 months, did any doctor, dentist, nurse, or other health professional advise you to quit smoking cigarettes or using any other tobacco products?

3: Assess: Determine willingness to quit or make a quit attempt.

Question: The last time a health professional advised you to quit using tobacco, did they also ask if you wanted to try to quit?

4: Assist: Aid the patient in quitting.

Question: The last time a health professional advised you to quit using tobacco, did they also offer any assistance, information, or additional advice to help you quit?

5: Arrange: Provide or schedule follow-up contact.

Question: Did they schedule any follow-up contacts, either in person or by phone, or arrange for someone else to call you to see how your quit attempt was going?

Note: The actual word order of the questions was: Advised, Asked, Assessed, Assisted, and Arranged; however, questions are presented in the standard 5 A’s order.

## Abbreviations

USPHS: U.S. Public Health Service Clinical Practice
NATS: National Adult Tobacco Survey
CASRO: Council of American Survey and Research Organizations
OR: odds ratio
CI: confidence interval
FDA: Food and Drug Administration

## References

[1] U.S. Department of Health and Human Services (2014). The Health Consequences of Smoking-50 Years of Progress: A Report of the Surgeon General.

[2] Jha P, Ramasundarahettige C, Landsman V, Rostron B, Thun M, Anderson RN (2013). 21st century hazards of smoking and benefits of cessation in the United States. N Engl J Med.

[3] Fiore MC, Bailey WC, Cohen SJ, Dorfman SF, Goldstein MG, Gritz ER (2008). Treating Tobacco Use and Dependence: 2008 Update—Clinical Practice Guideline.

[4] Maciosek MV, Coffield AB, Edwards NM, Flottemesch TJ, Goodman MJ, Solberg LI (2006). Priorities for improving utilization of clinical preventive services results. Am J Prev Med.

[5] Solberg LL, Maciosek MV, Edwards NM, Khanchandani HS, Goodman MJ (2006). Repeated tobacco-use screening and intervention in clinical practice health impact and cost effectiveness. Am J Prev Med.

[6] Kruger J, Shaw L, Kahende J, Frank E. Health care providers’ advice to quit smoking, National Health Interview Survey, 2000, 2005, 2010. Prev Chronic Dis. 2012;9:110310. DOI: http://dx.doi.org/10.5888/pcd9.110340. 10.5888/pcd9.110340 PMC3468305 22814236

[7] Rigotti NA (2011). Integrating comprehensive tobacco treatment into the evolving US health care system. Arch Intern Med.

[8] Shiffman S, Brockwell SE, Pillitteri JL, Gitchell JG (2008). Use of smoking-cessation treatments in the United States. Am J Prev Med.

[9] Centers for Disease Control and Prevention (2011). Quitting smoking among adults: United States 2001–2010. MMWR.

[10] Centers for Disease Control and Prevention (2011). 2009–2010 National Adult Tobacco Survey: Methodology Report.

[11] Council of American Survey Research Organizations (CASRO). Code of Standards and Ethics for Survey Research; 2011. https://c.ymcdn.com/sites/www.casro.org/resource/resmgr/casro_code_of_standards.pdf

[12] Gordon GB. Survey weight adjustment using PROC WTADJUST from SUDAAN V10. In Proceedings of the annual meeting of the American Association for Public Opinion Research: 13–16 May 2010; Chicago. https://www.rti.org/pubs/aapor10_brown_poster.pdf

[13] Ministry of Health (2014). The New Zealand Guidelines for Helping People to Stop Smoking.

[14] Kruger J, O’Halloran A, Rosenthal A (2015). Assessment of compliance with US Public Health Service Clinical Practice Guideline for tobacco by primary care physicians. Harm Reduct J.

[15] Quinn VP, Hollis JF, Smith KS, Rigotti NA, Solberg SI, Hu W (2009). Effectiveness of the 5-As tobacco cessation treatments in nine HMOs. J Gen Intern Med.

[16] Chase EC, McMenamin SB, Halpin HA (2007). Medicaid provider delivery of the 5A’s for smoking cessation counseling. Nicotine Tob Res.

[17] Quinn VP, Stevens VJ, Hollis JF, Rigotti NA, Solberg LI, Gordon N (2005). Tobacco-cessation services and patient satisfaction in nine nonprofit HMO’s. Am J Prev Med.

[18] Gordon JS, Lichtenstein E, Severson HH (2006). Tobacco cessation in dental settings: research findings and future directions. Drug Alcohol Rev.

[19] Haskard Zolnierek KB, DiMatteo MR (2009). Physician Communication and Patient Adherence to Treatment: A Meta-analysis. Med Care.

[20] Gordon JS, Andrews JA, Crews KM, Payne TJ, Severson HH (2007). The 5A’s vs 3A’s plus proactive quitline referral in private practice dental offices: preliminary results. Tob Control.

[21] King BA, Dube SR, Babb SD, McAfee TA (2013). Patient-reported recall of smoking cessation interventions from a health professional. Prev Med.

[22] Fu SS, van Ryn M, Sherman SE, Burgess DJ, Noorbaloochi S, Clothier B, et al.. Proactive tobacco treatment and population-level cessation. Intern Med 2014, doi:10.1001/jamainternmed.2014.177 10.1001/jamainternmed.2014.177 24615217

[23] Orleans CT, Woolf SH, Rothemich SF, Marks JS, Isham GJ (2006). The top priority: Building a better system for tobacco-cessation counseling. Am J Prev Med.

[24] The Guide to Community Preventive Services. Reducing tobacco use and secondhand smoke exposure. Atlanta; 2006 http://www.thecommunityguide.org/tobacco/index.html

[25] Centers for Disease Control and Prevention (2013). Impact of a national tobacco education campaign on weekly numbers of quitline calls and website visitors—United States, March 4-June 23, 2013. MMWR.

[26] Centers for Disease Control and Prevention (2014). Best Practices for Comprehensive Tobacco Control Programs—2014.

[27] Institute of Medicine (2007). Ending the Tobacco Problem: A Blueprint for the Nation.

[28] Schroeder SA (2005). What to do with a patient who smokes. JAMA.

[29] Fiore MC, Keller PA, Curry SJ (2007). Health systems changes to facilitate the delivery of tobacco-dependence treatment. Am J Prev Med.

[30] Centers for Medicare & Medicaid Services. Medicare National Coverage Determinations Manual: Chapter 1, Part 4 (Sections 200–310.1) Coverage Determinations; Washington, DC. https://www.cms.gov/Regulations-and-Guidance/Guidance/Manuals/downloads/ncd103c1_part4.pdf

[31] Centers for Medicare & Medicaid Services. Medicaid Increasing Coverage of Aids for Tobacco Cessation; Baltimore. https://www.medicaid.gov/Medicaid-CHIP-Program-Information/By-Topics/Benefits/Tobacco.html

[32] Patient Protection and Affordable Care Act, Public Law 111–148, U.S. Statutes at Large 2010;119:124.

[33] United States Department of Labor. FAQs about Affordable Care Act implementation (Part XIX); Washington. http://www.dol.gov/ebsa/faqs/faq-aca19.html

[34] Piper ME, Fiore MC, Smith SS, Jorenby DE, Wilson JR, Zehner ME (2003). Use of the vital sign stamp as a systematic screening tool to promote smoking cessation. Mayo Clin Proc.

[35] Lindholm C, Adsit R, Bain P, Reber PM, Brein T, Redmond L (2010). A demonstration project for using the electronic health record to identify and treat tobacco users. WMJ.

